# Robust Polymer Nanocomposite Membranes Incorporating Discrete TiO_2_ Nanotubes for Water Treatment

**DOI:** 10.3390/nano9091186

**Published:** 2019-08-21

**Authors:** Najia Mahdi, Pawan Kumar, Ankur Goswami, Basil Perdicakis, Karthik Shankar, Mohtada Sadrzadeh

**Affiliations:** 1 Department of Electrical and Computer Engineering, University of Alberta, Edmonton, AB T6G1H9, Canada; 2 Department of Materials Science and Engineering, Indian Institute of Technology Delhi, New Delhi 110016, India; 3 Suncor Energy Inc., P.O. Box 2844, 150-6th Ave. SW, Calgary, AB T2P3E3, Canada; 4 Department of Mechanical Engineering, University of Alberta, Edmonton, AB T6G1H9, Canada

**Keywords:** superhydrophilic nanotubes, electrochemical anodization, blend membranes, asymmetric polysulfone

## Abstract

Polyethersulfone (PES) is a polymeric permeable material used in ultrafiltration (UF) membranes due to its high thermomechanical and chemical stability. The hydrophobic nature of PES membranes renders them prone to fouling and restricts the practical applications of PES in the fabrication of water treatment membranes. The present study demonstrates a non-solvent-induced phase separation (NIPS) approach to modifying PES membranes with different concentrations of discrete TiO_2_ nanotubes (TNTs). Zeta potential and contact angle measurements showed enhanced hydrophilicity and surface negative charge in TNTs/PES nanocomposite membranes compared to unmodified PES membranes. To discern the antifouling and permeation properties of the TNTs/PES membranes, steam assisted gravity drainage (SAGD) wastewater obtained from the Athabasca oil sands of Alberta was used. The TiO_2_ modified polymer nanocomposite membranes resulted in a higher organic matter rejection and water flux than the unmodified PES membrane. The addition of discrete TNTs at 1 wt% afforded maximum water flux (82 L/m^2^ h at 40 psi), organic matter rejection (53.9%), and antifouling properties (29% improvement in comparison to pristine PES membrane). An enhancement in fouling resistance of TNTs/PES nanocomposite membranes was observed in flux recovery ratio experiments.

## 1. Introduction

Water sources, such as lakes, groundwater, and rivers, have been contaminated by industrial waste disposal directly or indirectly [1]. The coastal areas of seas and oceans have also witnessed a dramatic increase in contaminants [2]. The shortage of clean water continues to increase due to which improved water usage efficiency is of paramount importance. Many industrial processes that use steam production in boilers are completely reliant on the supply of fresh water. Therefore, it is important to treat wastewater using energy efficient and robust techniques to ensure a continuous supply of fresh water. Various methods have been explored for water recycling that are both more energy efficient and environmentally sustainable [3]. Non-reactive interfacial interaction between a solid surface and aqueous medium, i.e., sorption and filtration based on microstructure, electrostatics, dispersion forces, etc., can be utilized for the cost-effective and facile purification of water [4]. Filtration using polymeric membranes for wastewater has seen increased adoption recently due to cost efficiency, low energy consumption, reliability, and ease of contaminant removal without using harmful products and no phase change at room temperature operations in comparison to other methods of water recycling [5,6,7,8,9].

Polymers are economical materials that can be used for large-scale membrane fabrication and are easy to modify as needed [9,10,11,12,13]. Polyethersulfone (PES) is a commonly used polymeric material for filtration applications due to its excellent thermomechanical, chemical, and contaminant separation properties. However, the hydrophobicity of PES results in poor performance of membrane which could hinder its anti-fouling properties negating many of its advantages during the filtration process [12]. Such poor anti-fouling properties have a detrimental effect on the permeation properties which causes a decrease in lifespan of the membrane resulting in an increase of operation cost [14]. Surface modified ultrafiltration (UF) membranes have been explored significantly to enhance the anti-fouling properties of the PES membrane by increasing the hydrophilicity and decreasing the surface charge as these parameters can have significant positive effects on the initial stages of fouling [15,16]. A variety of methods such as chemical grafting [17], the addition of antifouling blending materials [13], surface coating [18] and plasma treatment [19] have been explored to fabricate anti-fouling surfaces.

Currently, much research and development are underway on the surface modification of membranes by the incorporation of hydrophilic nanoparticles into the PES polymer to fabricate polymer nanocomposite membranes with higher surface hydrophilicity, increased anti-fouling properties and enhanced separation performance [20,21]. Numerous studies investigated the effect of various nanostructures including porous nanostructures such as carbon nanotubes (CNTs), cellulose nanocrystals, graphene oxide nanoplates, zeolites, mesoporous silica, and nonporous metal oxide nanoparticles, such as TiO_2_, MgO, ZnO, Al_2_O_3_, ZrO_2_, Fe_2_O_3_, and SiO_2_, on the structural morphology, transport properties, and surface characteristics of the fabricated nanocomposite membranes [22,23,24,25,26,27,28]. Atomic layer deposition (ALD) of metal oxides on the polymeric membrane is an approach that provides hydrophilicity and antifouling properties to the filtration membranes [29]. Yang et al. reported that ~10 nm TiO_2_ and SnO_2_-deposited via ALD on polyvinylidene fluoride (PVDF) membranes exhibit excellent anti-crude-oil fouling performance [30].

Recently, TiO_2_ nanostructures have received significant attention as membrane additives due to their stability, non-toxicity, self-cleaning ability, and natural abundance [31,32].

TiO_2_ nanotubes are hollow annular structures synthesized by the electrochemical anodization process that can be tailored to have cylindrical or square pores, with tunable diameters from ~10–1000 nm [33,34]. These nanotubes have a high effective surface area for interfacial adhesion to the polymer due to nanoscopic wall roughness, and the availability of both inner and outer tube walls. In the present study, the effect of incorporation of different concentrations of discrete TiO_2_ NTs on the physicochemical characteristics and permeation properties of the polyethersulfone (PES) membranes are reported. In addition, bare TiO_2_ nanotubes are superhydrophilic and can be rendered superhydrophobic or amphiphobic if needed by suitable surface functionalization [35,36].

The non-solvent-induced phase separation (NIPS) method was used to fabricate TiO_2_ NT incorporated PES membranes. The morphology of structures, chemical compositions and surface properties of the synthesized membranes were investigated by field emission scanning electron microscopy (FESEM), atomic force Microscopy (AFM), Fourier-transform infrared spectroscopy (FTIR), surface zeta potential, and water contact angle measurements. Water flux, organic matter rejection, and fouling resistance of the TiO_2_ NTs incorporated PES membranes were studied using both real and synthetically-produced water, and were compared with unmodified PES membranes.

## 2. Materials and Methods

### 2.1. Chemical and Reagents

The ultrafiltration membranes were made using the PES polymer with a molecular weight of 58 kDa obtained from BASF (Ludwigshafen, Germany). PVP (molecular weight, MW: 360 kDa) was procured from Sigma Aldrich (St. Louis, MO, USA) and used as an additive. Potassium chloride (KCl) and N,N-dimethylacetamide (DMA) were purchased from Fisher Scientific (Waltham, MA, USA). The discrete TiO_2_ NTs were synthesized using an electrochemical anodization process followed by sonication for discretization. Diethylene glycol (DEG) and ammonium fluoride (NH_4_F) were purchased from Sigma Aldrich to be used as an electrolyte in electrochemical anodization. Octadecylphosphonic acid (ODPA) to functionalize discrete nanotubes was also indented from Sigma Aldrich. An industrial wastewater, produced from steam-assisted gravity drainage (SAGD) in the Athabasca oil sands industry (AB, Canada) was used for the membrane permeation and fouling characteristics tests. The produced water is the feed water to the warm lime softener (WLS) of current SAGD water treatment facilities.

### 2.2. Preparation of TiO_2_ NTs/PES Membranes

**Figure 1:**
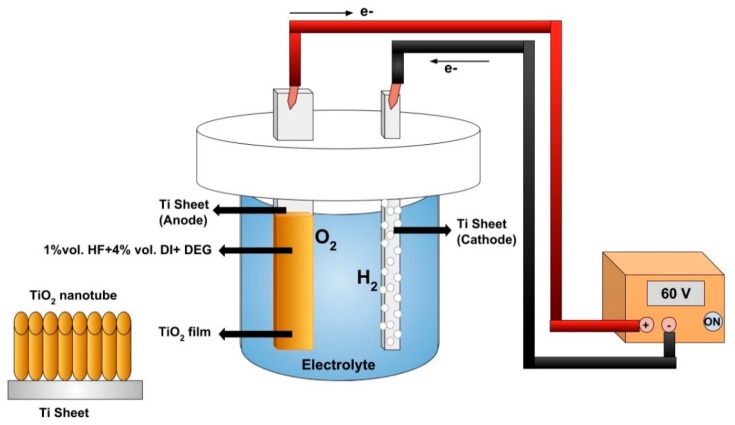
Electrochemical anodization process used to form TiO_2_ NTs.

**Figure 2:**
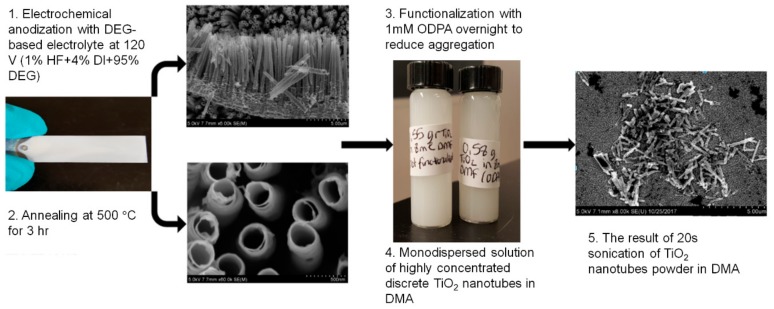
Discrete TiO_2_ NT synthesis, functionalization, and discretization methods explained.

The preparation of TiO_2_ NTs/PES membranes consisted of two steps. The first step includes synthesis of TiO_2_ NTs via an electrochemical anodization process in DEG based electrolyte using a two-electrode electrochemical cell as shown in Figure 1. The synthesized TiO_2_ NTs on foil were annealed in a furnace at 500 °C for 3 h followed by functionalization with 1 mM ODPA solution overnight (Figure 2). The TiO_2_ NTs were discretized in 8 mL DMA using a probe sonicator. Different concentrations of discrete NT solutions were made to test the effect of concentration of nanomaterials on PES membrane structure and permeation properties.

**Figure 3:**
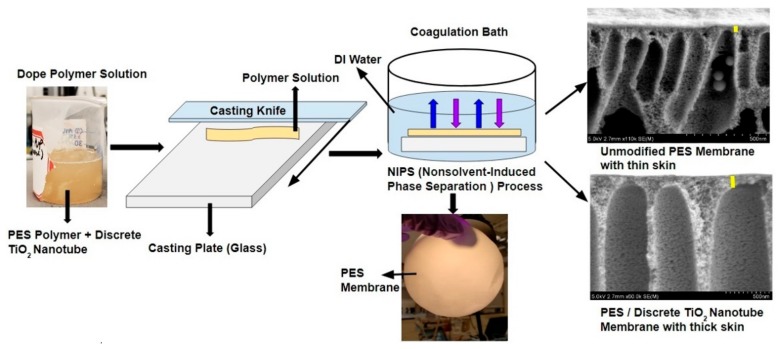
Fabrication process of TiO_2_ NTs/PES nanocomposite membranes.

The second step, which is shown in Figure 3, was to fabricate TiO_2_ NTs/PES nanocomposite membranes. First, a casting solution was prepared by dissolving desired amounts of PES pellets, discrete TiO_2_ NTs and PVP in DMA solvent. In this work, to produce porous polymer films, homogeneously doped polymer solutions were prepared by mixing DMA with 14 wt% PES, 2 wt% PVP, and various ratios of discrete TiO_2_ NTs to polymer (1, 0.5, and 0.25 wt%). Next, a probe sonicator was used to disperse the discrete TiO_2_ NTs in DMA uniformly. PVP and PES mixture was then added to the discrete TiO_2_ NT/DMA solution and stirred overnight at 100 rpm. The obtained mixture, which is also called dope solution, was kept at room temperature for 24 h to remove air bubbles from the casting solution and to produce a defect-free membrane. A film applicator (Gardco, MICROM II, Pompano Beach, FL, USA) with a clearance gap of 190 μm and a casting speed of 5 mm/s was used to cast the solution on a flat glass surface. The film was then immersed in DI water and the membrane formation was completed after 24 h by liquid-liquid demixing.

### 2.3. Measurements of Porosity and Pore Size

The average pore size of the membrane was measured using the following expression below which was proposed by Guerout–Elford–Ferry [37]:

(1) $$r_{m}=\sqrt{\frac{\left(2.9-1.75\varepsilon \right)8\eta lQ}{\varepsilon A\Delta P}}$$

where *Q* is the permeate volumetric flow rate (m^3^/s), *η* is the water viscosity which is 8.9 × 10^−4^ Pas at 25 °C, and Δ*P* is the transmembrane pressure (Pa). The applied transmembrane pressures in this study were 10, 20, 30, and 40 psi.

Average porosity of membranes which is the ratio of total pore volume to the volume of the membrane was calculated using the gravimetric method [18]. The following equation was used to calculate the porosity of a membrane. To calculate the average porosity of each membrane the measurements were repeated three times.

(2) $$\varepsilon =\frac{w_{1}-w_{2}}{Al\rho }$$

where *w*_1_ and *w*_2_ are mass of wet and dry membranes (g) respectively, *ρ* is the water density (0.997 g/cm^3^ at 25 °C), *l* is the membrane thickness (cm), and *A* is the surface area (cm^2^) of the membrane.

### 2.4. Pure Water Flux Measurement

A dead-end filtration cell setup consisted of a membrane with an area of 41.8 cm^2^ and a dead-end stirred cell (Millipore Amicon Ultra, UFSC40001, Burlington, MA, USA) with a capacity of 400 mL. To monitor and record the permeate water flux over time, a digital balance (ME 4002, Mettler Toledo, Columbus, OH, USA) connected to a computer was employed. Various transmembrane pressures were applied using the pressurized nitrogen gas. The solution in the dead-end cell, in this case, the deionized (DI) water was stirred at a rate of 80 rpm. The equation below was used to calculate the water flux (*J*_0_):

(3) $$J_{0}=\frac{W}{A\Delta t}$$

where *A* is the membrane effective area (m^2^), *W* is the mass of the permeate water (kg), and *t* is the permeation time (h).

### 2.5. Fouling Tests

The fouling tests consisted of a three-step experimental procedure. The pure water flux, *J_W_*_1_, was first measured. The, *J_WF_*, water flux during the filtration of WLS inlet water, was then recorded. Lastly, the pure water filtration after hydraulic washing of the membrane surface with DI water for 15 min was performed and the pure water flux of the cleaned membrane *J_W_*_2_ was measured again. Based on these fouling tests, the fouling evaluation parameters namely total flux decline ratio (*DR_t_*), the sum of the reversible flux decline ratio (*DR_r_*), irreversible flux decline ratio (*DR_ir_*), and flux recovery ratio (*FRR*) were calculated using the equations below [38]:

(4) $$DR_{t}=1-\frac{J_{wF}}{J_{w1}}$$

(5) $$FRR=\frac{J_{w2}}{J_{w1}}$$

(6) $$DR_{r}=\frac{\left(J_{w_{2}}-J_{wF}\right)}{J_{w1}}$$

(7) $$DR_{i}=1-\frac{J_{w2}}{J_{w1}}$$

### 2.6. Total Organic Carbon Estimation

The total organic carbon (TOC) in the WLS inlet water was measured using a TOC analyzer (Shimadzu, Kyoto, Japan, model TOC-V; detection range 3–25,000 mg/L). The TOC analyzer measures the level of the organic contaminants or organic molecules in purified water and in this work the concentration of organic carbon atoms covalently bonded in the organic molecules of the PES based membranes was detected by the TOC analyzer. This parameter can be used to test the efficiency of the treatment process. The rejection of the organic matter can also be calculated using the measured TOC concentrations of the permeate (*C_p_*) and feed (*C_F_*) solutions. The rejection was calculated using the equation below:

(8) $$R=\left(1-\frac{C_{p}}{C_{F}}\right)\times 100$$

### 2.7. Chemical Composition Test (FTIR)

To verify the functionalization of titanium nanotube arrays (TNTAs) with ODPA, the IR active vibrations were recorded on a Digilab (Varian, Palo Alto, CA, USA) FTS 7000 FT-Infrared Spectrophotometer equipped with a UMA 600 Microscope and ZnSe ATR accessory. The sample was scratched from the surface and deposited directly on the ZnSe crystal and nitrogen gas flow was maintained through the ATR assembly. The spectra were accumulated by averaging 64 scans in the frequency range of 850–4000 cm^−1^.

### 2.8. X-ray Photoelectron Spectroscopy (XPS)

X-ray photoelectron spectroscopy (XPS) was used to determine the surface and subsurface (up to ~10 nm) chemical composition and oxidation state of materials using an Axis-Ultra, Kratos Analytical Limited instrument (Trafford Park, Manchester, UK) equipped with a monochromatic Al-Kα source (15 kV, 50 W) with photon energy of 1486.7 eV under ultrahigh vacuum (~10^−8^ Torr). The binding energy of adventitious carbons C1s peak at ≈ 284.8 eV was used as standard (carbon correction) to assign the peak of other elements. The raw spectra in vms format were deconvoluted into various peak components using CasaXPS software (version 2.3.22) and exported data was plotted in origin 2018.

### 2.9. Surface Properties (Wettability and Surface Charge)

Surface wetting characteristics of the fabricated membranes with various concentrations of discrete TiO_2_ NTs were measured using FTA-200 contact angle analyzer (First Ten Ångstroms, Portsmouth, VA, USA). DI water was used as a drop method to measure the contact angle. The lower the wettability of the membrane, the higher is the contact angle.

The Surpass3 zeta potential analyzer (Anton Paar GmbH, Graz, Austria) was used to analyze the charging behavior of the membrane surface when in contact with a liquid such as DI water. The surface zeta potential is derived from a flow of potential which arises from the interaction between the motions of liquid relating to the solid surface. The zeta potential values were obtained using various pH values (5, 6, 7, and 8) at 25 °C using 0.001 M KCl solution.

### 2.10. Membrane Morphology Study

The morphologies and cross-sections of the dried membranes were studied using the field emission scanning electron microscopy (Hitachi S-4800 Field Emission SEM, FE-SEM, Tokyo, Japan). The conductivity of the membranes was improved by sputtering 10 nm of gold on the surface of the membrane using Denton Gold Sputterer Unit (for SEM). A high vacuum with 5 kV/20 µA condition was used to image the cross-sectional structure of the prepared nanocomposite membranes.

### 2.11. Atomic Force Microscopy

Atomic force microscopy (AFM, Bruker, The Dimension Fast Scan Atomic Force Microscope, Santa Barbara, CA, USA) was used to image the surface topography of the TiO_2_ NTs/PES nanocomposite membrane.

## 3. Results

### 3.1. FTIR Measurement Results

**Figure 4:**
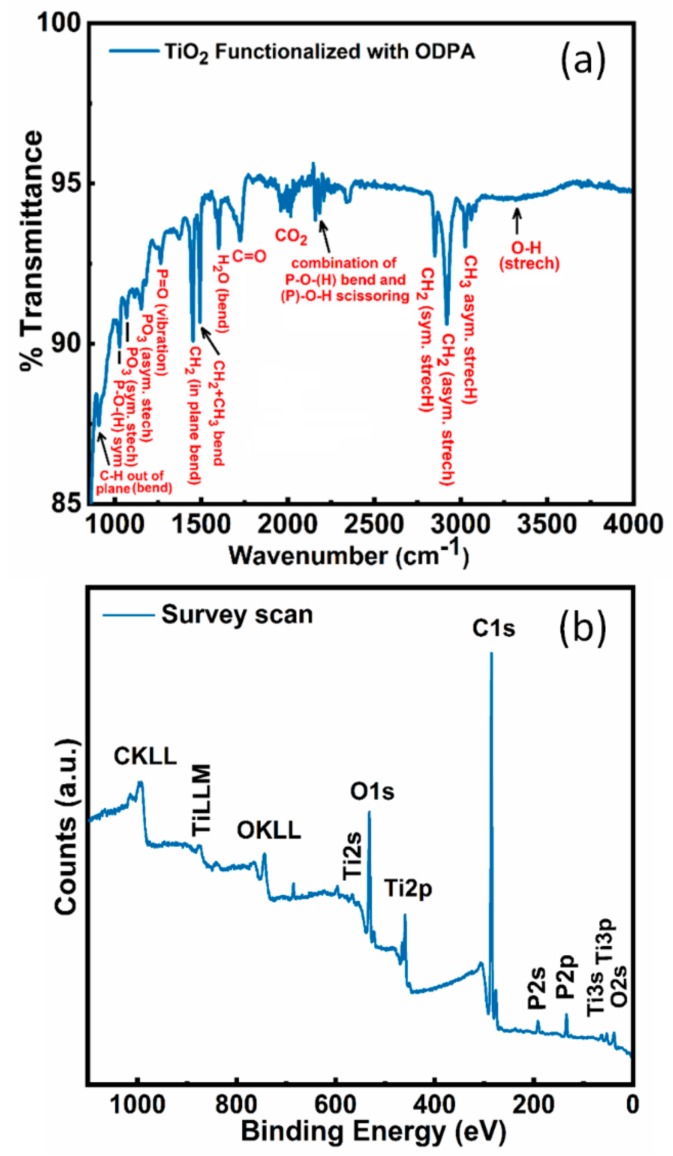
(**a**) FTIR spectra of ODPA functionalized TiO_2_ NTs; (**b**) XPS elemental survey scan of ODPA functionalized TiO_2_ nanotubes.

A vibrational spectrum of ODPA monolayer functionalized TiO_2_ nanotubes was collected to validate the successful chemical functionalization of TiO_2_ NTs surface with ODPA molecules (Figure 4a). The FTIR spectrum of ODPA functionalized TiO_2_ exhibits a characteristic IR absorption band at 2848 and 2917 cm^−1^ due to symmetric and anti-symmetric C–H stretching vibrations of CH_2_ in alkyl chain of ODPA [35,36,39]. The IR peak at 3019 cm^−1^ was derived from C–H stretch of terminal methyl (–CH_3_) groups. Further, the peak observed at 3320 and 1605 cm^−1^ was assigned to O–H stretch and bending vibration of surface adsorbed water, respectively. Additionally, various peaks corresponding to symmetric and asymmetric bending vibration of CH_2_ and CH_3_ groups in ODPA were observed at 1492 cm^−1^ (CH_2_ + CH_3_ bend), 1450 cm^−1^ (CH_2_ in-plane bend) and 906 cm^−1^ (C–H out of plane bend) confirming the presence of ODPA molecule on the surface of TiO_2_ nanotubes. The IR spectrum of ODPA functionalized TNTAs in the frequency range 850–1300 cm^−1^ shows various IR bands at 1265, 1152, 1065 and 1027 cm^−1^ assigned to P=O vibration, PO_3_ asymmetric stretch, PO_3_ symmetric stretch and P–O–(H) symmetric stretch respectively. The presence of these IR bands clearly demonstrates successful chemical functionalization of TNTA with OPDA via the reaction between the –OH group on the surface of TiO_2_ and the phosphonate group (–H_2_PO_4_) on ODPA to form a phosphonate ester linkage (ROPO_2_–). TiO_2_ nanotubes were functionalized with the 18-carbon long ODPA to improve the monodispersity of the discrete TiO_2_ NTs in DMA (since the long alkyl chains prevent agglomeration of individual nanotubes). It is also well-known that organophosphonate monolayers bind very strongly to TiO_2_ surfaces and are not vulnerable to hydrolytic desorption [40].

### 3.2. XPS Results

**Figure 5:**
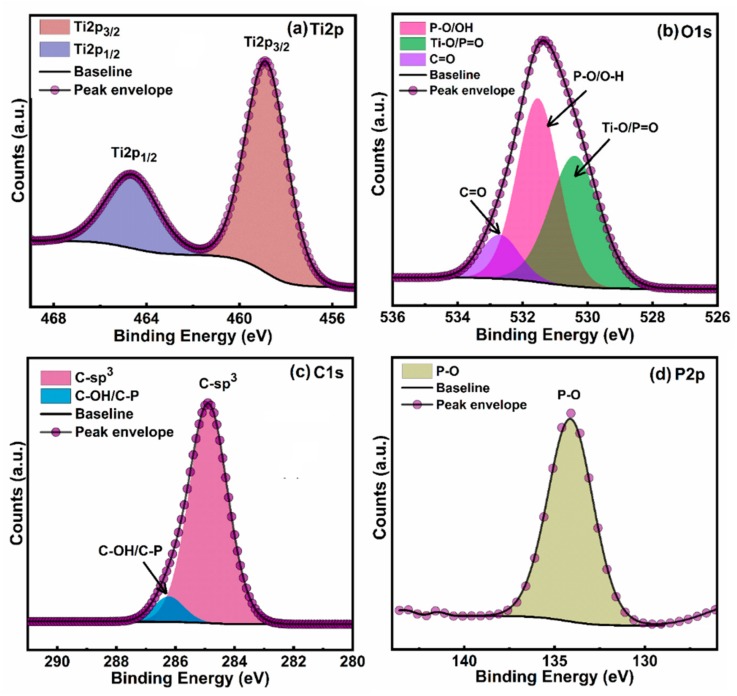
Core-level HR-XPS spectra of ODPA functionalized TiO_2_ nanotubes in (**a**) Ti2p region; (**b**) O1s region; (**c**) C1s region; and (**d**) O1s region.

The oxidation state and surface chemical attributes of ODPA functionalized TiO_2_ nanotubes were determined using X-ray photoelectron microscopy (XPS) (Figure 4b and Figure 5a–d). The appearance of C1s, O1s, Ti2p, P2p peaks in XPS elemental survey scan of ODPA functionalized TiO_2_ nanotubes demonstrate successful functionalization of TiO_2_ nanotubes with carbon and phosphorous rich organic molecules. The signal intensity of C1s peak was much higher, agreeing with a high concentration of carbon at the surface of TiO_2_ due to better coverage with ODPA monolayer (Figure 5b). High-resolution XPS spectra (HR-XPS) of ODPA functionalized TiO_2_ nanotubes in the Ti2p region show two peaks components located at a binding energy (*BE*) 458.8 and 464.6 eV, originated due to Ti2p_3/2_ and Ti2p_1/2_ peak components of the Ti^4+^ state in the TiO_2_ crystal lattice (Figure 5a) [41]. The peak separation of 5.8 eV between Ti2p_3/2_ and Ti2p_1/2_ peaks and their respective positions validate the O^2−^ coordinated Ti^4+^ in tetragonal anatase phase TiO_2_ [42]. Core-level HR-XPS spectra of ODPA functionalized TiO_2_ nanotubes in O1s region can be deconvoluted into three peak components centered at *BE* ≈ 530.4, 531.5 and 532.7 eV (Figure 5b). The peak component at *BE* value 530.4 eV was originated from the contribution from Ti-coordinated oxygen atoms (O^2−^–Ti^4+^ forming TiO_6_ octahedron) present in the crystal lattice of TiO_2_ and P=O of surface-adsorbed ODPA molecule while an intense peak at *BE* ≈ 531.5 eV was corroborated to non-lattice adventitious oxygen atoms (–OH groups) and P–O oxygen of ODPA molecule (Figure 5b) [35,43]. Relatively small peak component at 532.7 eV was assigned to adventitious carbon bonded oxygen atoms (C=O). Deconvoluted HR-XPS spectra of ODPA functionalized TiO_2_ nanotubes in the C1s region gave two peak components, at 284.8 and 286.3 eV (Figure 5c). The major peak component at 284.8 eV originated from sp^3^ hybridized carbon atoms composing the alkyl chain of ODPA while the less intense shoulder peak at 286.3 eV was arose from the contribution of C–OH/C–P type carbon atoms [1,44]. Additionally, a single peak at *BE* ≈134.1 eV, in the HR-XPS of ODPA-grafted TiO_2_ nanotubes in the P2p region was assigned to phosphorous in the phosphonate group (P–O) bonded to TiO_2_ and confirms successful grafting of ODPA on TiO_2_’s surface (Figure 5d) [45].

### 3.3. Surface and Cross-Section Morphology

**Figure 6:**
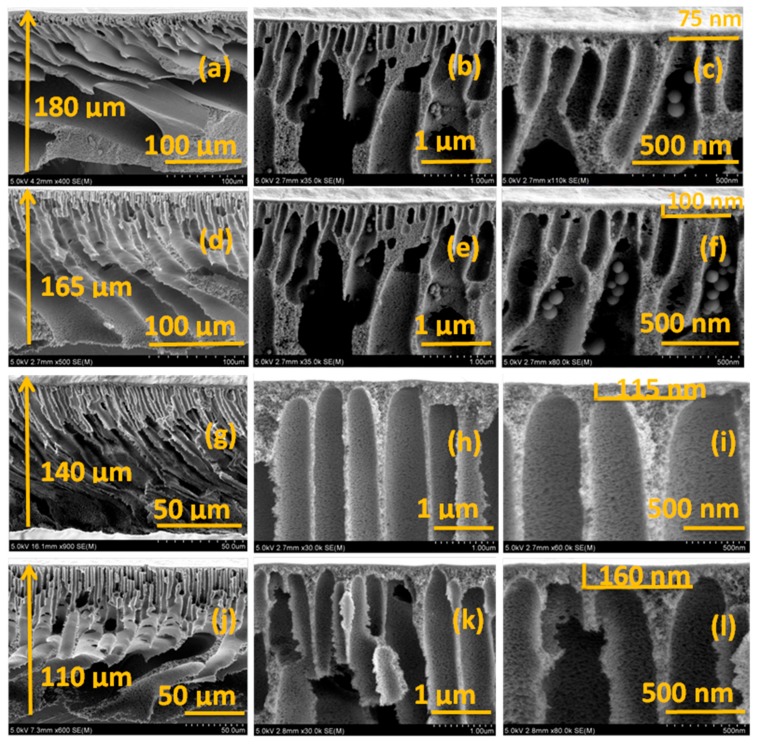
The cross-sectional SEM images of pristine PES and TiO_2_ NTs/PES nanocomposite membranes (**a**–**c**); pristine PES membrane (**d**–**f**); 0.25% TiO_2_/PES membrane (**g**–**i**); 0.5% TiO_2_/PES membrane; and (**j**–**l**) 1% TiO_2_/PES membrane.

The membranes were immersed in liquid nitrogen for a few seconds to get a clear cut before preparing the samples for the internal cross-sectional imaging. The images of the unmodified PES membrane and the TiO_2_ NTs incorporated PES membranes are presented in Figure 6 for a comparison. The PES membranes used for microfiltration (MF) and UF purposes have a common trait which is a porous finger-type asymmetric structure consisting of macrovoids and a top dense skin layer [46]. Figure 6 shows this very clearly in all the membrane cross-sectional images. According to Figure 6, the addition of discrete TiO_2_ NTs to the unmodified PES membrane decreased the thickness of the membrane. However, the average skin thickness of the membranes increased as the concentration of discrete TiO_2_ NTs increased. A possible explanation is that the addition of the discrete NTs slows down the solvent/anti-solvent exchange rate in the coagulation bath and this causes the formation of thinner membranes with a thicker skin layer due to the entrapment of more discrete TiO_2_ NTs at the top surface during phase separation [38]. The discrete TiO_2_ NTs tend to enhance thermodynamic instability of the casting solution that accelerates the exchange of solvent and non-solvent [46]. The additional volume of TiO_2_ nanotubes, together with the existence of some portion of the hollow internal nanotube that is not infiltrated by the polymer, is expected to produce swelling of the polymer film by hydrophilic nanotubes before its solidification, thus allowing more non-solvent to flow into the casting film during NIPS process which, in turn, tends to increase the thickness of the membrane. However, an increase in the viscosity of the casting solution due to addition of discrete TiO_2_ NTs, together with the excellent miscibility of the PDPS-coated TiO_2_ NTs with PES, reversed these effects. As a result, the mutual diffusivities between non-solvent and solvent were reduced causing a decrease in the membrane thickness.

### 3.4. Atomic Force Microscopy (AFM)

**Figure 7:**
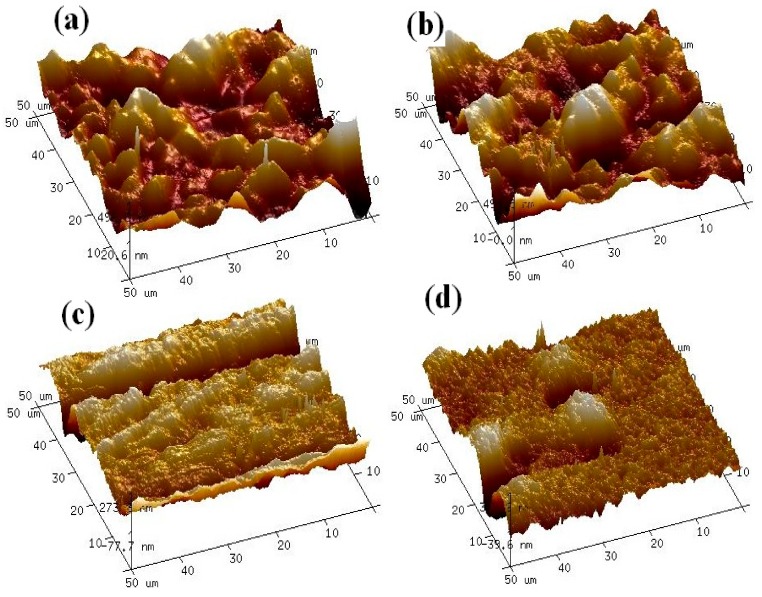
Topography of discrete TiO_2_-based PES membranes using atomic force microscopy: (**a**) 1% TiO_2_/PES membrane; (**b**) 0.5% TiO_2_/PES membrane; (**c**) 0.25% TiO_2_/PES membrane; and (**d**) pristine PES membrane.

Roughness is a direct metric of surface topography which can be measured with AFM. According to the images in Figure 7, the surface roughness increased with increasing the concentration of discrete TiO_2_ NTs in PES membranes. The surface roughness of pristine PES, 0.25% TiO_2_/PES, 0.5% TiO_2_/PES, and 1% TiO_2_/PES were measured to be 57.1, 83.3, 121, and 133 nm, respectively. The PES membrane with 1% discrete TiO_2_ NTs had the highest surface roughness. This membrane also had the lowest contact angle meaning that a part of improved wettability of nanocomposite membranes can be attributed to the increased roughness. According to the Wenzel equation, a membrane that is slightly hydrophilic becomes more hydrophilic as the surface roughness increases. In addition, based on Table 1, 1% TiO_2_/PES membrane was more negatively charged (−32.0 ± 0.6 mV) which proves the intended material improvement, e.g., addition of TiO_2_ NTs does increases the hydrophilicity of the membranes. 

### 3.5. Contact Angle Measurement Results

Water contact angle analysis is a technique to study the hydrophilic properties of synthesized membranes. In general, a higher contact angle means that the membrane is more hydrophobic, and a lower contact angle reveals more hydrophilicity and higher surface energy of the membrane. A syringe which was positioned above the sample surface was used to deposit water droplets with a volume of 1 µL and a high-resolution camera was used to capture the side image of the droplet. The angle of the image was then analyzed using a protractor to measure the contact angles. For each membrane, three different measurements were taken from various spots on the membrane and the average values were reported. The contact angles of the membranes are presented in Table 1. The contact angle of the bare PES membrane was 65 ± 2° and as the concentration of discrete TiO_2_ NTs increased from 0.25% to 1%, the contact angle was observed to decrease from 49 ± 1° to 37 ± 1°. The contact angle of the bare TiO_2_ NTs was measured to be 8.0 ± 2°, which is highly hydrophilic compared to the PES membrane. The accumulation of hydrophilic discrete TiO_2_ NTs on the surface during phase inversion process reduced the interface energy. Based on contact angle measurements, the addition of discrete TiO_2_ NTs to PES membranes leads to the formation of more hydrophilic membranes. More hydrophilicity of microfiltration (MF)/ultrafiltration (UF) membranes can be correlated with their antifouling properties, as will be discussed later.

### 3.6. Membrane Surface Charge Results

The nature of surface charge on the membrane is a crucial parameter which governs the hydrophilicity and longevity of the membrane. The incorporation of discrete TiO_2_ NTs in TiO_2_ NTs/PES increases the hydrophilicity due to the raising the surface free energy which concomitantly reduces fouling of the PES membranes. The contact angle measurements and the surface charge of the membranes are presented in Table 1. The PES polymer consists of a polyetherphenylsulfone backbone with pendant sulfonate (–SO_3_H) groups and the number of sulfonic acid groups is dependent on the degree of sulfonation. At pH values higher than the isoelectric point (IEP), the sulfonate groups in PES polymer get dissociated into negatively charged sulfate ions. Due to low dissociation constant (pKa), the sulfonate groups readily get dissociated in water at pH-7, which gives them surface negative charge as observed from zeta potential measurements. In the pristine PES material, a certain fraction of sulfonate functionalities remains undissociated due to strong hydrogen bonding and inaccessibility of solvent. Upon the incorporation of discrete TiO_2_ NTs, the membrane surface becomes more accessible to solvents which facilitates dissociation of sulfonic acid groups and increases negative change. Additionally, the surface of TiO_2_ possesses plenty of hydroxyl groups (–OH) bonded with Ti atoms. These hydroxyl groups get deprotonated at elevated pH which further contributes to surface negative charge of the membrane. The PES membrane with 1 wt% TiO_2_ NTs is the most negatively charged in this study (see Table 1), and is expected to reduce the fouling by organic and inorganic contaminants in the water due to electrostatic repulsion [47].

### 3.7. Permeability of Membranes

**Figure 8:**
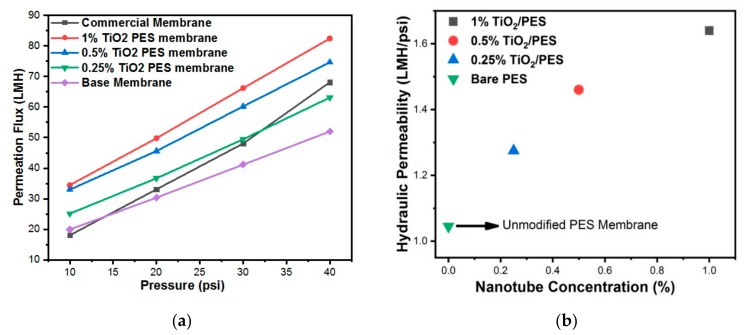
(**a**) Permeation flux as a function of transmembrane pressure; and (**b**) hydraulic permeability of membranes as a function of NT concentration.

In asymmetric membranes, there are two layers; the top dense layer which governs the permeation properties and the bottom porous layer which provides mechanical strength. The addition of the inorganic discrete TiO_2_ NTs which are polar attract the polar water molecules to the membrane surface which leads to an improvement in pure water flux. The pure water flux graph of pristine PES membrane and nanocomposite PES membranes with 0.25 to 1 wt% loading of discrete TiO_2_ NTs, as a function transmembrane pressure is shown in Figure 8a. The hydraulic permeability of the PES based membranes was obtained from the slopes of this figure. As can be seen in this figure, incorporating discrete TiO_2_ NTs has resulted in higher water permeability than unmodified PES membrane. The addition of more hydrophilic hydroxyl groups to the surface of the membrane improved the hydrophilicity (Table 1) which also increased water permeability of the membrane [48]. The nanocomposite membrane prepared by 1 wt% discrete TiO_2_ NTs was found to provide the maximum hydraulic permeability of 1.65 L/m^2^h(LMH)/psi. The extracted hydraulic permeability results from Figure 8a are shown in Figure 8b. As can be seen in this figure, increasing the concentration of discrete NTs up to 1 wt% increased the water permeability. This result can be attributed to a remarkable increase in hydrophilicity of membranes (Table 1). In addition, the overall thickness of membranes was reduced by the incorporation of TiO_2_ NTs (Figure 6), which decreased the resistance of the membranes against water transport.

### 3.8. Porosity and Mean Pore Radius of Membrane

**Figure 9:**
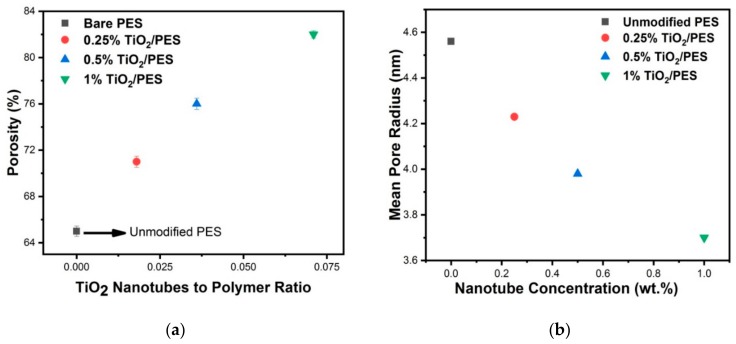
(**a**) Porosity and (**b**) mean pore radius plots of various membranes.

The porosity (%) is the void space over the total volume of a polymer matrix and is one of the most important properties of a polymer. The pore size and pore volume are essential parameters to be measured prior to the application of a polymer in various fields. Porosity is measured by filling the pores either with a liquid or gas. Following the gravimetric method [49], the wet and dry masses of various membranes were measured, and this is shown in Table 2. The porosity of nanocomposite membranes is shown in Figure 9. By the addition of discrete TiO_2_ NTs up to 1 wt%, the overall porosity is increased. Theoretically, PVP as an additive to PES increases the thermodynamic instability due to its non-solvent property and kinetic hindrance due to its high molecular weight. As result, this relation between kinetic hindrance and thermodynamic enhancement of the casting solution affects the solidified membranes’ functional and structural properties by changing the porosity of phase inversion membranes [46,50]. The addition of hydrophilic discrete TiO_2_ NTs to the polymer solution further decreases its thermodynamic stability. Addition of both PVP and discrete TiO_2_ NTs results in thermodynamic variation which enhances the demixing of solution, thus leading to the formation of more porous structures. On the other hand, an increase in the viscosity of casting solution by the addition of TiO_2_ NTs can lead to the delayed demixing of solvent and non-solvent and thus formation of denser structures. This implies the presence of an optimum loading of discrete NTs (0.25 to 1 wt% TiO_2_ NTs) for improving the water flux. The increase in porosity is another parameter that improved the water flux results as shown in Figure 8. According to Figure 9, the mean pore size decreased, and the overall porosity increased as the percentage of discrete TiO_2_ NTs in membrane increased (see Equation (1)). The reduced pore diameter can potentially improve the separation properties of the nanocomposite membranes. As mentioned before, the separation properties of membranes are governed by the overall porosity and the membrane thickness and the addition of higher concentration of NTs increases both of these parameters, thus bettering the membrane properties for water treatment applications. In the present study, the main reason for the increase in the removal of organic matter by the addition of 1 wt% discrete TiO_2_ NTs, as will be explained in the next section, can be due to the increase in the thickness of skin layer (FESEM images in Figure 6).

### 3.9. Separation Performance of Membranes

The separation performance of nanocomposite membranes was evaluated by the filtration of WLS feed water. Table 3 presents the organic matter rejection results. A better performance for the removal of organic matter from oil sands produced water was observed for the nanocomposite membranes when compared with pristine membrane. The TOC rejection increased from 11.7% (unmodified PES membrane) to more than 50% for the 1 wt% discrete TiO_2_ NTs/PES nanocomposite membrane. Based on the data presented in Figure 4 and Table 3, the addition of 1 wt% discrete TiO_2_ NT has maximized both water flux (82 LMH at 40 psi) and TOC rejection (53.9%). The rejection was calculated using equation 8 in Section 2.5.

### 3.10. Fouling Characteristics of Membranes

The fouling characteristics of unmodified pristine PES membrane and discrete TiO_2_ NTs/PES nanocomposite membranes during the filtration of WLS feed water are shown in Table 3. To investigate the effect of TiO_2_ NTs on flux decline over time, all experiments were conducted at a similar initial permeation flux. Additionally, in all experiments, the feed solution chemistry, temperature, and hydrodynamic of membrane modules were kept similar. This allowed attributing the fouling propensity of membranes to the surface properties rather than feed properties and hydrodynamic conditions (e.g., drag force) [47,51]. The membranes were compressed at high pressure (70 psi) before the experiments to ensure that the flux decline over time is only due to the fouling of the membranes. At a constant initial flux, the flux decline can be attributed to the hydrophilicity, surface charge, and roughness of the modified membranes [52].

**Figure 10:**
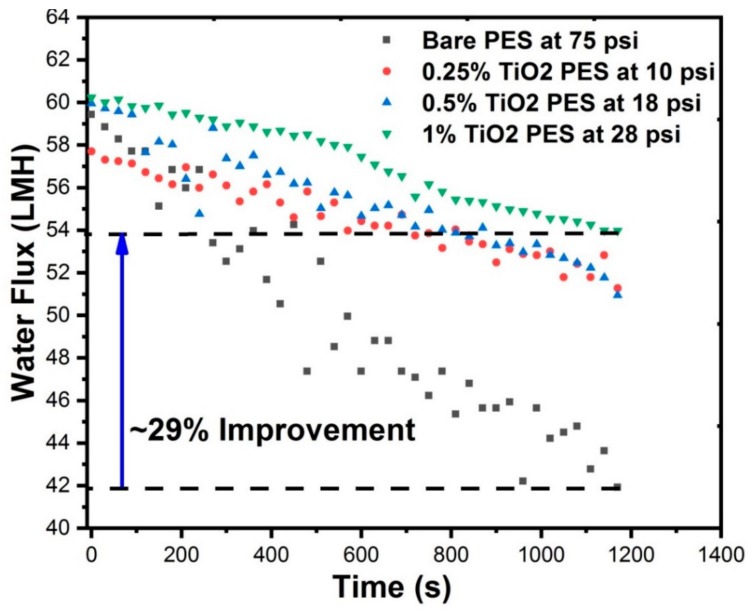
Water flux vs. time discrete TiO_2_ nanocomposite membranes and unmodified PES membrane due to fouling.

According to the Figure 10, the flux decline of the unmodified membrane is more than the PES membranes modified with discrete TiO_2_ NTs. The PES membrane with 1 wt% discrete TiO_2_ NTs showed approximately 29% more water flux than the unmodified PES membrane after 20 min of filtration. It is well known that the membranes with more negative surface charge and higher hydrophilicity have less interaction amongst the functional dissolved organic compounds in the feed solution and the polar groups on the membranes surface. This can be explained by formation of a water layer on the surface of the membrane that hinders foulant attachment to the membrane surface due to the formation of hydrogen bonds among the surface hydrophilic groups to start with. Due to the hydrophobic nature of most organic matter in the fluids tested [51], the fouling materials are less inclined to attach to a hydrophilic surface where there is smaller hydrophobic interaction with membrane surface. Membrane surface roughness, characterized by AFM, also plays a significant role in fouling. Foulant materials are entrapped in the edgy zones, behind the peaks and consequently clog the valleys on the surface of MF/UF membranes resulting in significant decrease in permeate flux. In this study, the combination of the hydrophilic surface of TiO_2_ NTs-based nanocomposite membranes along with their high surface potential (Table 1) seem to have dominated the roughness effect and made them less prone to fouling by dissolved organic matter, which can be beneficial for oil sands-produced water treatment.

**Figure 11:**
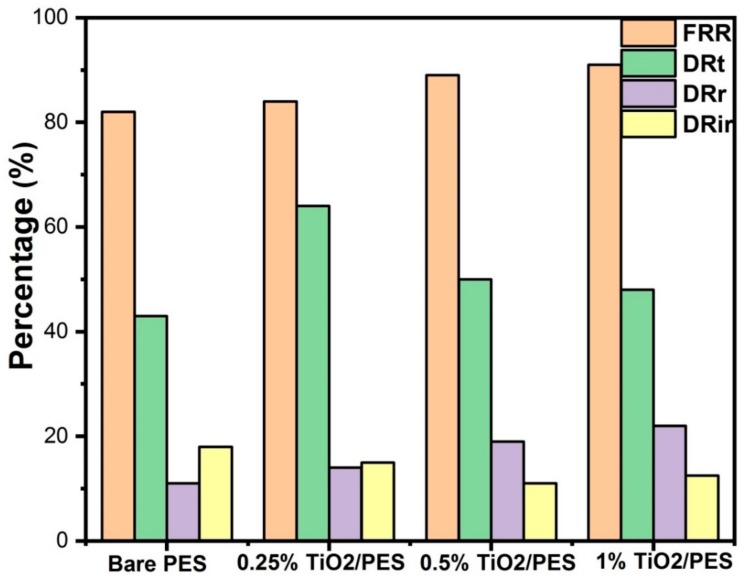
Comparisons of fouling characteristics of unmodified PES and PES/discrete TiO_2_ nanocomposite membranes.

The total flux decline ratio (*DR_t_*), irreversible fouling ratio (*DR_ir_*), water flux recovery ratio (*FRR*), and reversible fouling ratio (*DR_r_*) for the pristine PES membrane and TiO_2_ NTs/PES nanocomposite membranes are shown in Figure 11. The membranes showed flux decline during the filtration of WLS feed water for 20 min, which was possibly due to the existence of inorganic and organic materials on the surface of membranes. TiO_2_ NTs with high surface area are great adsorbent materials that adsorb foulants on the surface of the membrane thus decreasing the irreversible flux reduction. The *DR_ir_* was found to be 12.5%, 11%, and 15% for 1, 0.5, and 0.25 wt% TiO_2_ loading as compared to 18% for unmodified PES membrane. The 1 wt% TiO_2_/PES membrane indicated a 9% greater flux recovery ratio than the pristine PES membrane, suggesting an improvement in antifouling characteristics of the unmodified membrane by the incorporation of TiO_2_ NTs.

## 4. Conclusions

In this study, discrete TiO_2_ NTs with various concentrations were blended into a polymeric membrane matrix via the NIPS technique. The surface properties of the nanocomposite membranes such as negative surface charge and hydrophilicity have noticeably changed to be stronger. The contact angle and zeta potential measurements showed the fabrication of more hydrophilic and negatively charged TiO_2_ NTs PES nanocomposite membranes. All discrete TiO_2_ NTs incorporated PES membranes showed better organic matter rejection and water flux when compared with the pristine PES membrane. The incorporation of discrete TiO_2_ NTs, up to 1 wt% enhanced the water flux due to an increase in overall porosity and hydrophilicity of the membranes. The most favorable loading to improve both organic matter rejection and water flux was found to be 1 wt% TiO_2_ NTs incorporated PES membranes which removed 53.9% of dissolved organic matter from SAGD-produced water. The SAGD WLS feed water was used to test the fouling propensity of membranes. The results showed that by the addition of discrete TiO_2_ NTs, the fouling tendency of the membranes has hindered due to improved surface properties. Discrete TiO_2_ NTs at its optimal loading (1 wt%) has provided the maximum rejection of organic matter (53.9%), water flux (82 LMH at 40 psi), and antifouling properties (29% improvement compared to pristine PES membrane). The flux recovery ratio (FRR) experiments have confirmed notable improvement in the antifouling property of discrete TiO_2_ NTs incorporated PES membranes.

As mentioned before, one of the potential benefits of TiO_2_ NTs incorporated PES membranes is their low cost compared to other nanomaterials due to their nearly endless lifetime. The low human toxicity, and high thermal and chemical stability are also other main advantages of TiO_2_ NTs which make them a good material in water filtration processes commercially. This membrane technology can easily be implemented into an existing commercial production facility due to facile synthesis methods of TiO_2_ NTs as well as the discrete TiO_2_ NTs. Since it is possible to produce discrete nanotubes in large scale, commercial fabrication of nanotube nanocomposite PES membranes will be an easy solution to fouling of membranes in water treatment.

## Figures and Tables

**Table 1:** Surface charge/roughness of PES membranes and contact angle measurement results.

Membranes	Surface Roughness (nm)	Contact Angle	Zeta Potential (mV) at pH 5	Zeta Potential (mV) at pH 6	Zeta Potential (mV) at pH 7	Zeta Potential (mV) at pH 8
Unmodified PES	57.1	65.1 ± 2 °	−24.2 ± 0.1	−26.6 ± 0.7	−27.5 ± 0.1	−28.0 ± 0.9
Unmodified TiO_2_ NT membrane	–	8.0 ± 2 °	−8.3 ± 0.5	−8.8 ± 0.4	−9.2 ± 0.2	−10.1 ± 0.8
PES with 0.25 wt% TiO_2_ NTs	83.3	49.1 ± 1 °	−25.2 ± 0.8	−27.0 ± 0.6	−27.8 ± 0.4	−28.4 ± 0.5
PES with 0.5 wt% TiO_2_ NTs	121	40.5 ± 2 °	−26.4 ± 0.7	−27.3 ± 0.4	−28.6 ± 0.6	−29.7 ± 0.8
PES with 1 wt% TiO_2_ NTs	133	37.4 ± 1 °	−28.2 ± 1	−29.4 ± 0.4	−31.5 ± 0.3	−32.0 ± 0.6

**Table 2:** Measured masses of dry and wet membranes for porosity and mean pore radius.

Membrane	Mass of Dry Membrane (mg)	Mass of Wet Membrane (mg)
Unmodified PES	14.6 ± 1	502.1 ± 2
PES with 0.25 wt% TiO_2_ NTs	25.0 ± 2	557.5 ± 3
PES with 0.5 wt% TiO_2_ NTs	27.2 ± 2	597.2 ± 1
PES with 1 wt% TiO_2_ NTs	24.2 ± 2	639.2 ± 2

**Table 3:** Removal of organic matter from WLS inlet water (TOC: 500 ppm).

	Permeate TOC (mg/L)	Permeate TC (mg/L)	Permeate IC (mg/L)	WLS Feed Water Rejection (%)
Bare PES	441.28	468.74	27.46	11.7
1 wt% TiO_2_/PES	230.70	253.80	23.10	53.9
0.5 wt% TiO_2_/PES	413.51	447.40	33.90	17.3
0.25 wt% TiO_2_/PES	424.23	451.20	26.97	15.15
